# The Human Liver-Expressed Lectin CD302 Restricts Hepatitis C Virus Infection

**DOI:** 10.1128/jvi.01995-21

**Published:** 2022-03-17

**Authors:** Birthe Reinecke, Nicola Frericks, Chris Lauber, Katja Dinkelborg, Alina Matthaei, Florian W. R. Vondran, Patrick Behrendt, Sibylle Haid, Richard J. P. Brown, Thomas Pietschmann

**Affiliations:** a Institute for Experimental Virology, TWINCORE Centre for Experimental and Clinical Infection Research, a joint venture between Medical School Hannover (MHH) and Helmholtz Centre for Infection Research (HZI), Hannover, Germany; b Cluster of Excellence RESIST (EXC 2155), Hannover Medical School, Hannover, Germany; c Department for General, Visceral and Transplant Surgery, Hannover Medical School, Hannover, Germany; d German Center for Infection Research (DZIF), Partner Site Hannover-Braunschweig, Hannover, Germany; e Division of Veterinary Medicine, Paul Ehrlich Institute, Langen, Germany; University of Southern California

**Keywords:** hepatitis C virus, hepatitis E virus, lectin, mechanism, polymorphism, transcript variant

## Abstract

C-type lectin domain-containing proteins (CTLDcps) shape host responses to pathogens and infectious disease outcomes. Previously, we identified the murine CTLDcp *Cd302* as restriction factor, limiting hepatitis C virus (HCV) infection of murine hepatocytes. In this study, we investigated in detail the human orthologue’s ability to restrict HCV infection in human liver cells. *CD302* overexpression in Huh-7.5 cells potently inhibited infection of diverse HCV chimeras representing seven genotypes. Transcriptional profiling revealed abundant *CD302* mRNA expression in human hepatocytes, the natural cellular target of HCV. Knockdown of endogenously expressed *CD302* modestly enhanced HCV infection of Huh-7.5 cells and primary human hepatocytes. Functional analysis of naturally occurring *CD302* transcript variants and engineered *CD302* mutants showed that the C-type lectin-like domain (CTLD) is essential for HCV restriction, whereas the cytoplasmic domain (CPD) is dispensable. Coding single nucleotide polymorphisms occurring in human populations and mapping to different domains of *CD302* did not influence the capacity of *CD302* to restrict HCV. Assessment of the anti-HCV phenotype at different life cycle stages indicated that CD302 preferentially targets the viral entry step. In contrast to the murine orthologue, overexpression of human *CD302* did not modulate downstream expression of nuclear receptor-controlled genes. Ectopic *CD302* expression restricted infection of liver tropic hepatitis E virus (HEV), while it did not affect infection rates of two respiratory viruses, including respiratory syncytial virus (RSV) and the alpha coronavirus HVCoV-229E. Together, these findings suggest that *CD302* contributes to liver cell-intrinsic defense against HCV and might mediate broader antiviral defenses against additional hepatotropic viruses.

**IMPORTANCE** The liver represents an immunoprivileged organ characterized by enhanced resistance to immune responses. However, the importance of liver cell-endogenous, noncytolytic innate immune responses in pathogen control is not well defined. Although the role of myeloid cell-expressed CTLDcps in host responses to viruses has been characterized in detail, we have little information about their potential functions in the liver and their relevance for immune responses in this organ. Human hepatocytes endogenously express the CTLDcp CD302. Here, we provide evidence that CD302 limits HCV infection of human liver cells, likely by inhibiting a viral cell entry step. We confirm that the dominant liver-expressed transcript variant, as well as naturally occurring coding variants of CD302, maintain the capacity to restrict HCV. We further show that the CTLD of the protein is critical for the anti-HCV activity and that overexpressed CD302 limits HEV infection. Thus, CD302 likely contributes to human liver-intrinsic antiviral defenses.

## INTRODUCTION

Hepatitis C virus is a plus-strand RNA virus of the family *Flaviviridae*. It is distributed globally and chronically infects an estimated 71 million people (1). Acute HCV infection progresses to chronicity in 55 to 85% of cases, leading to approximately 400,000 deaths in 2015, which were mostly caused by liver cirrhosis or hepatocellular carcinoma (1; https://www.who.int/en/news-room/fact-sheets/detail/hepatitis-c). Despite the development of highly effective direct-acting antivirals (DAAs), treatment availability is not universal, and reinfection after cure is still possible (2–4). Moreover, HCV transmission rates remain high, and a prophylactic vaccine is not available (5).

HCV has evolved multiple mechanisms to avoid immune control and establish viral persistence. These include, among others, viral interference with innate immune signaling. Pattern recognition receptors such as retinoid acid-inducible gene I (RIG-I), melanoma differentiation-associated protein 5 (MDA5), Toll-like receptor 3 (TLR3), and protein kinase R (PKR) are key cellular receptors recognizing HCV replication-derived double-stranded RNA triggering these antiviral responses. However, the HCV NS3-4A protease cleaves mitochondrial antiviral signaling protein (MAVS) and Toll-like receptor adaptor molecule 1 (TICAM1 or TRIF), thus blunting antiviral signaling via RIG-I, MDA5, and TLR3, respectively. In addition, the HCV NS5A phosphoprotein may inhibit PKR-dependent antiviral innate immunity (6, 7). Nevertheless, *ex vivo* infection studies with primary human hepatocytes, combined with *in vivo* data from infected chimpanzees and humans, have shown that HCV infection is accompanied by induction of vigorous innate immune responses (8–10), characterized by strong and broad induction of multiple interferon (IFN)-stimulated genes (ISGs) (11–13). Thus, viral signaling interference in infected cells may be incomplete. Alternatively, or in addition, these responses may be driven by activation of noninfected cells and/or by additional signaling pathways not inactivated by HCV. In any case, innate immune activation likely plays a critical role in HCV pathophysiology, as human polymorphisms within the IFN-λ locus are associated with natural HCV clearance and responses to IFN-based treatment. Moreover, many IFN-induced cellular effector proteins have been described, which blunt HCV replication (14–19).

Recently, we conducted a cDNA library screen to uncover murine factors that prevent HCV infection of mouse liver cells and discovered *Cd302* (synonyms, *Dcl-1* and *Clec13a*), a C-type lectin-like domain-containing protein (CTLDcp), as an HCV restriction factor in murine hepatocytes (20). Moreover, we noted a potential relevance of human *CD302* for limiting HCV infection of human liver cells, as ectopic expression of *CD302* inhibited HCV infection of Huh-7.5 hepatoma cells. In both human and murine hepatocytes, mRNA expression of *CD302*/*Cd302* was not modulated by treatment with either interferon or a double-stranded RNA (dsRNA) mimic (20).

Kato et al. and Lo et al. previously characterized the expression and function of human and murine *CD302* in immune cells (21, 22). Their work suggests that CD302 is important for migration of dendritic cells and endocytic and phagocytic antigen uptake, as well as for cell adhesion. Additional studies on *CD302* focus on its importance for different kinds of cancer (23–25). Lo et al. also noted abundant expression of *CD302* in liver cells (22). However, the function of liver cell-expressed *CD302* and, more generally, of CTLDcps expressed in hepatocytes remains unclear. In this study, we examined the relevance of ectopic and endogenously expressed *CD302* during HCV infection of human hepatoma Huh-7.5 cells and explanted primary hepatocytes.

## RESULTS

### Human *CD302* restricts infection of chimeric HCV representing seven genotypes.

**FIG 1:**
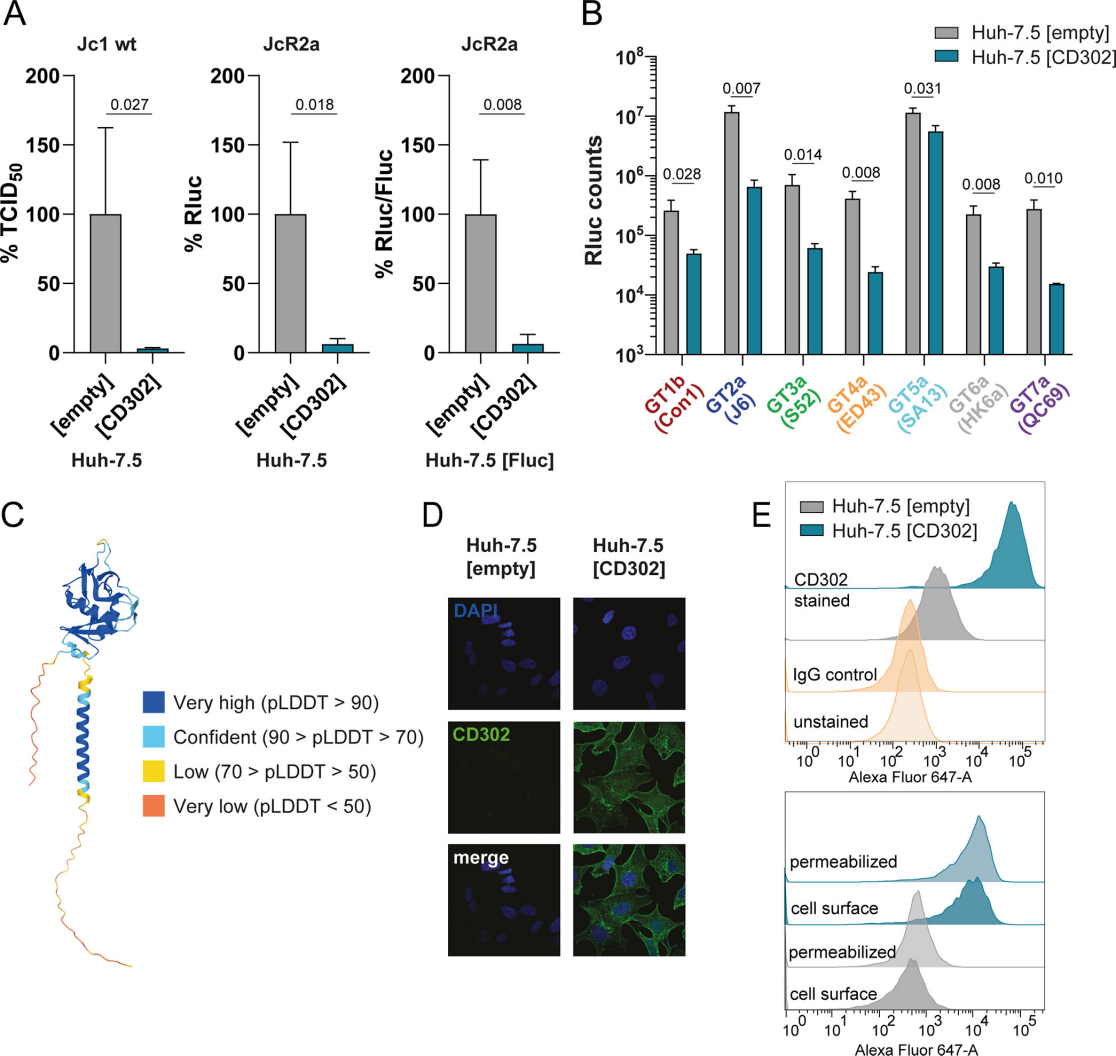
Ectopic expression of *CD302* restricts infection by HCV chimeras representing genotypes 1 to 7. (A, Left) Huh-7.5 cells stably overexpressing *CD302* or the empty control vector were infected with Jc1 wild type (WT), a GT2a J6-JFH1 chimera. Supernatants of these cells were collected 72 h postinfection (hpi), and infectious viruses present in these supernatants were titrated by using a limiting dilution assay. (A, Middle) The same cell lines were infected with an HCV *Renilla* luciferase reporter virus (JcR2a), and *Renilla* luciferase (Rluc) counts were determined at 72 hpi. Huh-7.5 cells ectopically expressing a firefly luciferase (Fluc) gene and stably overexpressing *CD302* or the empty control vector were infected with JcR2a and Fluc, and Rluc counts were determined at 72 hpi (A, Right). Data represent means and standard deviation (SD) of three independent biological replicates normalized to the empty vector control. *P* values are shown and calculated with one-tailed *t* test for each panel. TCID_50_/mL, 50% tissue culture infectious dose. (B) Huh-7.5 cells stably overexpressing *CD302* or an empty control vector were infected with luciferase-expressing HCV chimeras (GT 1 to 7), and Rluc counts were determined at 72 hpi. Data represent means and SD of three biological replicates. *P* values are shown as determined by multiple *t* tests with correction for multiple comparisons (FDR [*q*] < 0.05 for each comparison). The background of the assay was defined as mean of uninfected cells at 1.5 × 10^3^ Rluc counts. (C) Protein structure of CD302 as predicted by the AlphaFold model (48). pLDDT, predicted local distance difference test. (D and E) Detection of CD302 protein expression in given cell lines as determined by confocal microscopy (D) and FACS (E). (E, Top) CD302 protein expression at the cell surface with different controls. (E, Bottom) Comparison between cell surface staining and total cellular expression measured in detergent-permeabilized cells.

To build on our previous observation that human *CD302* may inhibit HCV infection of human liver cell lines (20), we conducted additional experiments involving authentic HCV cell culture (HCVcc) particles (Jc1 chimera) and JcR2a *Renilla* reporter viruses. Ectopic expression of CD302 significantly reduced *de novo* infectious Jc1 virus production from inoculated cells (Fig. 1A, left) and also significantly decreased infectivity of JcR2a reporter viruses in two different cell lines (Fig. 1A, middle and right). However, HCV is an exquisitely diverse human pathogen with viral strains mapping to eight distinct viral genotypes, which differ from each other by 30 to 35% at the nucleotide level (26, 27). Therefore, to explore the general relevance of human *CD302* for HCV infection, we quantified its antiviral effect using a panel of intergenotypic HCV reporter viruses representing HCV genotypes 1 to 7 (Fig. 1B). These chimeric viruses encode the structural proteins from isolates representing HCV genotypes 1 to 7 and the nonstructural proteins from the Japanese fulminant hepatitis 1 (JFH1) strain (28, 29). Ectopic expression of *CD302* in Huh-7.5 cells was confirmed by fluorescence-activated cell sorter (FACS) analysis and significantly reduced HCV infection rates for all tested virus chimeras, as evidenced by 2- to 18-fold reduced luciferase reporter gene signals at 72 h postinfection (hpi) (Fig. 1B). Thus, ectopic expression of *CD302* in a human hepatoma cell line limits HCV infection with a broad range of recombinant HCV chimeras expressing the structural proteins representing seven major HCV genotypes.

### *CD302* is preferentially surface expressed in hepatocytes.

**FIG 2:**
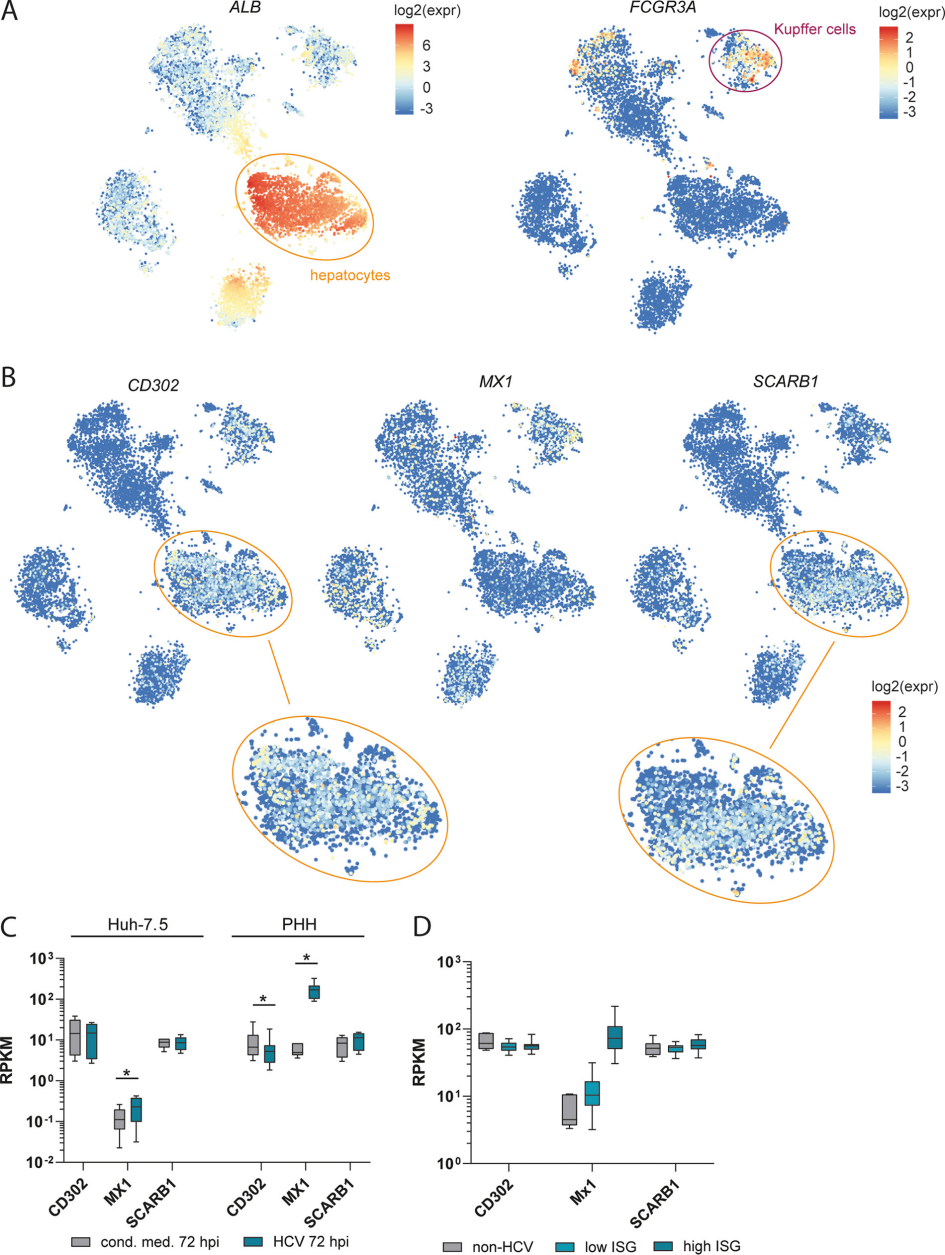
Endogenous and HCV-dependent mRNA expression of *CD302* in primary human liver cells. (A and B) T-distributed stochastic neighbor-embedding (t-SNE) plots highlighting mRNA expression of given genes across all cells of healthy human liver tissue (31). The color of each cell represents the gene expression according to the corresponding legend as log_2_ value of the expression. (A) Orange and magenta circles depict hepatocyte and Kupffer cell compartments, respectively (as defined previously [31]). (A, Left) ALB gene expression, a hepatocyte marker. (A, Right) *FCGR3A* gene expression, a marker for Kupffer cells. (B, Top) t-SNE plots depict *SCARB1*, *MX1*, and *CD302* mRNAs with single cell resolution. (B, Bottom) Hepatocyte compartments of *SCARB1* and *CD302* expression plots are enlarged. (C) Bulk transcriptional profiling of Huh-7.5 cells and PHHs. Box plot shows the expression of given mRNAs in Huh-7.5 cells and PHHs at 72 hpi with HCV (GT2a J6-JFH1 chimera Jc1) or upon treatment with conditioned medium (cond.med.) (13). *, FDR *P* < 0.05, determined by CLC Workbench (Qiagen). RPKM, reads per kilobase per million. (D) Gene expression levels in liver biopsy samples. Liver biopsy specimens derived from patients infected with HCV or non-HCV controls (32). HCV-infected samples were grouped into samples of low or high IFN-stimulated gene (ISG) responders depending on the expression of an ISG subset.

The predicted CD302 protein consists of a CTLD (PDB ID 2NAN), a transmembrane domain (TM), and a cytoplasmic tail (CPT) (Fig. 1C, top to bottom, and as previously published [20, 30]). Immunofluorescence staining of Huh-7.5 cells ectopically expressing CD302 revealed protein accumulation at the plasma membrane and some localization at the perinuclear compartment (Fig. 1D). FACS analysis of permeabilized and unpermeabilized Huh-7.5 cells, with or without ectopic CD302 expression, suggested endogenous expression of CD302 protein at the cell surface (Fig. 1E). To determine whether *CD302* is naturally expressed in the human liver and specifically in human hepatocytes, we examined *CD302* mRNA expression across all cells mapped in the human liver cell atlas, which is based on single cell-resolved transcriptional profiling of nine healthy human donors (31) (Fig. 2A and B). Albumin (*ALB*) was abundantly expressed in one large subcluster of the map, demarcating hepatocytes. A second subcluster was characterized by a strong Fc fragment of IgG receptor IIIa (*FCGR3A*) mRNA expression, thus identifying Kupffer cells (Fig. 2A). We noted high levels of *CD302* mRNA expression in many cells mapping to these two subclusters. *CD302* mRNA expression in hepatocytes was rather heterogeneous, ranging from very low to high mRNA levels, similar to that observed for *SCARB1* mRNA, which encodes an essential HCV cell entry factor (Fig. 2B). Expression was generally much higher than that of *MX1* mRNA, which represents an IFN-regulated gene. Using a data set generated earlier (13), we next investigated whether HCV infection of primary human hepatocytes (PHHs) or Huh-7.5 cells modulates endogenous *CD302* mRNA expression. While *MX1* expression was significantly upregulated in both PHHs and Huh-7.5 cells upon HCV infection at 72 hpi, *CD302* mRNA abundance was essentially unchanged in Huh-7.5 cells and significantly downregulated, with an average fold change of about −1.6 in PHHs (false-discovery rate [FDR] *P* = 0.0057) (Fig. 2C). Using data sets from liver biopsy specimens of chronically HCV-infected patients (32), we did not observe clear changes in *CD302* gene expression compared to non-HCV-infected patient controls, independent of a low or high ISG response to HCV (Fig. 2D). Again, *MX1* was upregulated in both groups. In conclusion, these data indicate that *CD302* mRNA is endogenously expressed in most hepatocytes in human liver tissue. Furthermore, these data are in accordance with previously published data that the *CD302* gene is not IFN regulated in murine or human hepatocytes (20).

### Endogenously expressed human *CD302* limits HCV infection of primary human hepatocytes.

**FIG 3:**
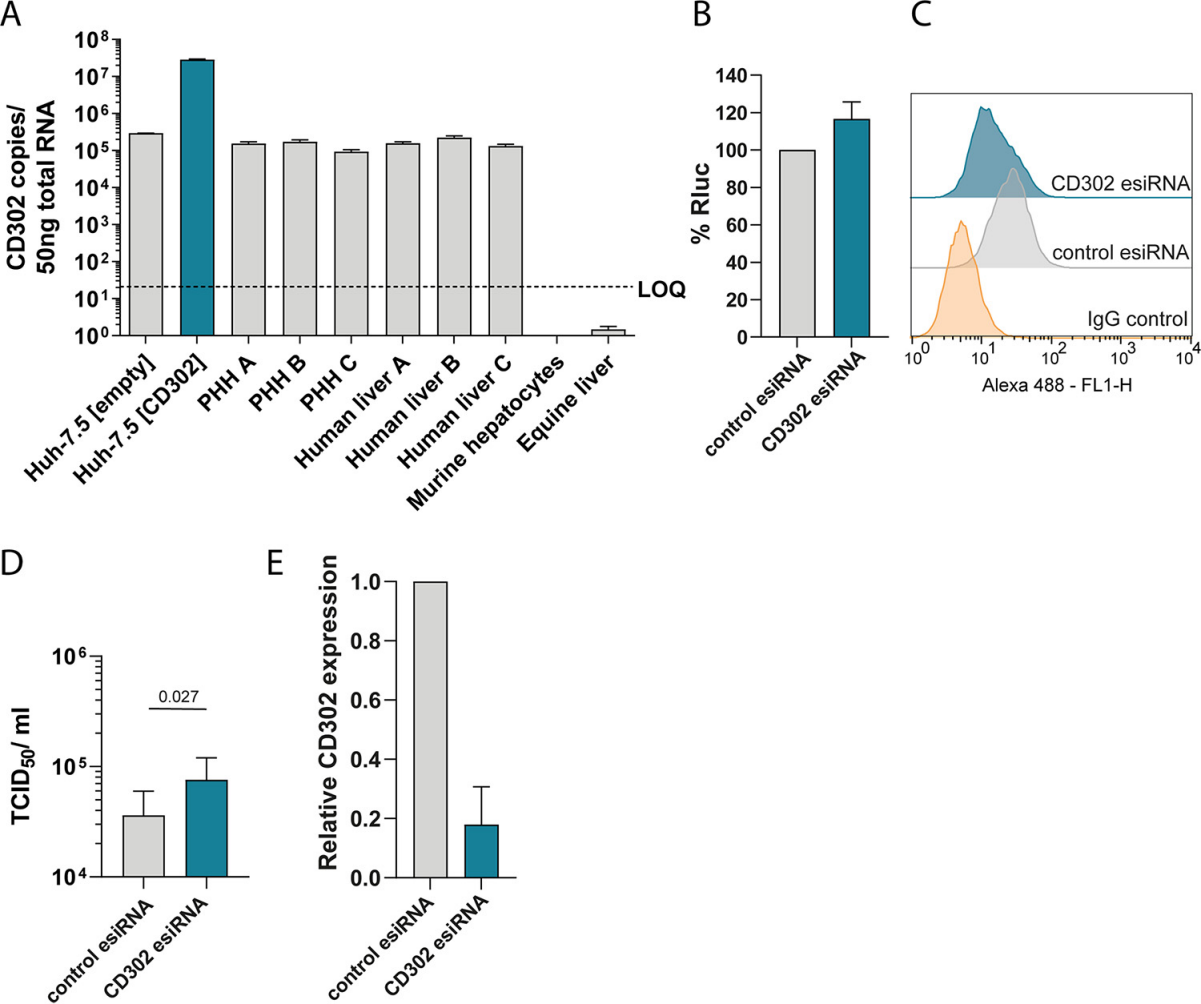
Endogenously expressed CD302 limits HCV infection of human hepatocytes. (A) Absolute quantification of *CD302* transcript copy numbers in cell lines, PHHs, and total liver. RNA from murine hepatocytes and equine liver was used as controls for primer specificity. A plasmid containing the *CD302* open reading frame (ORF) was serially diluted to generate a standard curve for RT-qPCR. (B) CD302 silenced or control esiRNA-treated Huh-7.5 cells were infected with the GT2a J6 JcR2a reporter virus. Luciferase activity was measured 48 h later and is shown relative to the values detected in control esiRNA-treated infected cells. Means and SD of three biological replicates are given. Cells were pretreated with esiRNAs for 48 h before inoculation. (C) FACS analysis of CD302 cell surface expression 48 h after transfection with given esiRNAs. Cells stained with the control IgG antibody instead of an anti-CD302 antibody served as control. (D and E) *CD302* silencing followed by HCV infection of primary human hepatocytes (PHHs). Ruxolitinib-treated PHHs were transfected with CD302 targeting or control esiRNAs for 48 h and were subsequently infected with Jc1 wt at an MOI of 1 to 1.4 for 72 h. (D) *De novo* infectious virus production into the culture fluid was quantified by using a limiting dilution infection assay with Huh-7.5 cells as target cells. Mean values of TCID_50_/mL of experiments with four independent human donors are given. *P* values shown above bars were determined by one-tailed *t* test from *n* = 4 biological replicates. (E) Relative *CD302* mRNA expression was measured by RT-qPCR. Cell lysates were collected at the time point of virus inoculation (48 h post-esiRNA transfection). Relative *CD302* expression was calculated by the 2^−ΔΔ^*^CT^* method.

We noted that Huh-7.5 cells express modest levels of CD302 at the cell surface and that the surface expression of CD302 is enhanced more than ca. 10-fold by lentiviral transduction (Fig. 1E). To compare these CD302 expression levels with the ones endogenously expressed in human hepatocytes, we determined the abundance of the *CD302* mRNA in PHHs from three different donors, as well as in liver tissue of three different donors. Expectedly and congruent with the FACS analysis (Fig. 1E), lentiviral gene transfer much enhanced *CD302* mRNA expression in Huh-7.5 cells (Fig. 3A). In contrast, untransduced Huh-7.5 (empty) cells displayed *CD302* mRNA expression comparable to the one in PHHs and in human liver tissue (Fig. 3A).

To test if endogenously expressed *CD302* restricts HCV, we first silenced *CD302* by RNA interference in Huh-7.5 cells and infected these cells with the GT2a J6 JcR2a reporter virus. The transfected endoribonuclease-prepared small interfering RNAs (esiRNAs) modestly decreased CD302 cell surface expression at the time point of infection and slightly enhanced HCV infection compared to control-treated cells (Fig. 3B and C). Next, we silenced *CD302* in PHHs and quantified HCV infection efficiency by measuring release of infectious viral progeny 72 h after inoculation of cells (Fig. 3D and E). PHHs mount a vigorous IFN-dependent innate immune response, which limits HCV infection (33, 34). Therefore, we treated these cells with ruxolitinib, an inhibitor of JAK/STAT signaling to enhance HCV infection rates and increase the measuring window of the assay (13). This was possible because human *CD302* expression is not affected by IFN treatment (20) (Fig. 2). Using this experimental system, RNA interference reduced *CD302* mRNA expression ca. 10-fold and concomitantly enhanced HCV infectious virus production ca. 2-fold compared to cells treated with control esiRNAs (Fig. 3D and E). Taken together, these data suggest that endogenously expressed *CD302* limits HCV infection of authentic target cells.

### Rare, natural *CD302* gene coding variants restrict HCV infection.

**FIG 4:**
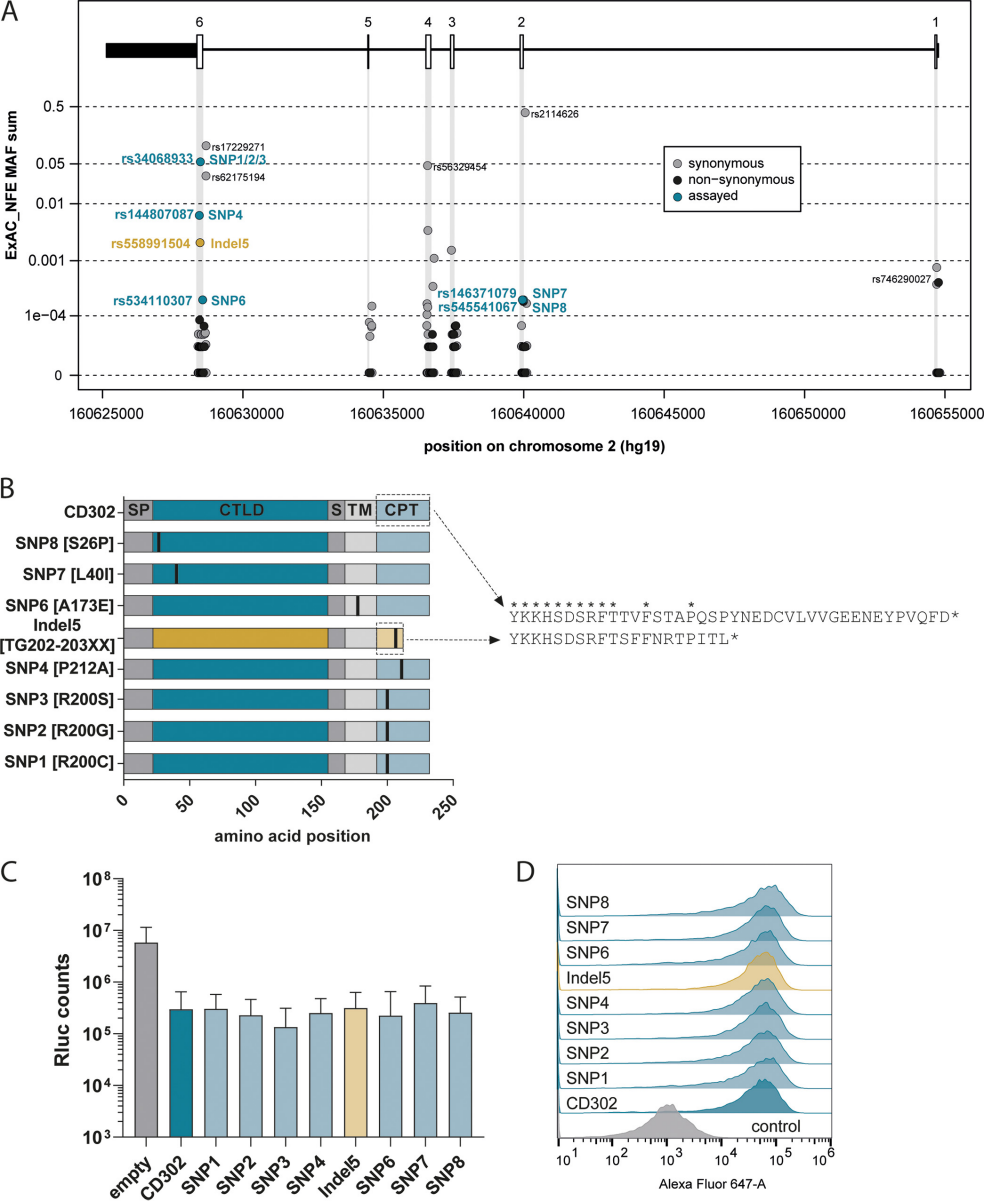
Impact of natural *CD302* genetic variation on HCV restriction. (A) Overview of SNPs in the Exome Aggregation Consortium (ExAC) cohort. Gene schematic visualizes the location of intron-exon boundaries in the hg19 genome assembly. Gray, black, or blue circles mark SNPs at their genome position and depending on their minor allele frequency (MAF). All assayed SNPs represent nonsynonymous mutations. (B) Amino acid changes induced by assayed SNPs. Brackets next to the SNP number refer to the amino acid change and its position in the encoded protein. Vertical black lines highlight locations of SNP-induced mutations in the protein. SP, signal peptide; CTLD, C-type lectin domain; S, spacer; TM, transmembrane domain; CPT, cytoplasmic tail. Right panel displays CPT amino acid sequences of wild type (top) compared to indel 5 (bottom). (C) Comparable HCV restriction ability of CD302 variants and the wild-type protein. Cells ectopically expressing the indicated variants were infected with GT2a J6 JcR2a for 72 h, and luciferase signal was measured. (D) Protein expression in overexpressing cells analyzed by FACS.

Next, we explored the influence of naturally occurring genetic variation in the protein coding regions of the *CD302* gene on HCV restriction. To this end, we used the Exome Aggregation Consortium database (ExAC database) to determine the minor allele frequencies (MAFs) of coding and noncoding single nucleotide polymorphisms (SNPs) mapping to exons of *CD302* in the non-Finish European population (Fig. 4A). The human *CD302* gene consists of six exons and is located on chromosome 2. In general, *CD302* is highly conserved, and MAFs of variants are low. We selected coding polymorphisms with an MAF of ≥0.0002 and a high likelihood for altering protein function for functional follow-up. We judged the potential impact of a given variant on protein function using Sorting Intolerant From Tolerant (SIFT), Polymorphism Phenotyping (PolyPhen), and combined annotation-dependent depletion (CADD) scores (Table 1, as presented in ENSEMBL [http://www.ensembl.org]). The CADD score is a measure of the predicted deleteriousness of a protein coding variant, with a score above 20 representing a high likelihood of disruption of function. Of note, we included all three variants for rs34068933, as the position displayed the highest variation within the population, and rs746290027 (exon 1, nonsynonymous) was predicted with low impact on protein function. In total, we analyzed eight previously identified allelic variants, which met our inclusion criteria (Fig. 4A). Two of these variants carried coding polymorphisms within the CTLD of CD302 (SNP7 and SNP8, respectively), and one variant harbored an SNP in the transmembrane domain (SNP6), whereas all other selected variants carried changes within the CPT of the protein (Fig. 4B). Notably, rs558991504 (indel 5) exhibits a deletion of two nucleotides, thus changing the reading frame of CD302 after amino acid 203 and leading to the addition of eight alternative downstream amino acids followed by a premature stop. All of these *CD302* variants were individually cloned into a lentiviral expression vector and used to generate Huh-7.5 cells overexpressing each variant. After antibiotic selection, we infected these cells with the GT2a J6 JcR2a reporter virus and measured luciferase activity 72 h later. Although bioinformatic prediction tools suggested an impact on protein function, all assayed variants were expressed in Huh-7.5 cells and restricted HCV infection similarly to wild-type CD302 (Fig. 4C and D). Collectively, even rare, naturally occurring CD302 coding variants with high predicted deleteriousness maintain an anti-HCV phenotype. Therefore, the HCV-limiting function of CD302 is highly conserved among allelic variants naturally occurring in human populations.

**TABLE 1:**
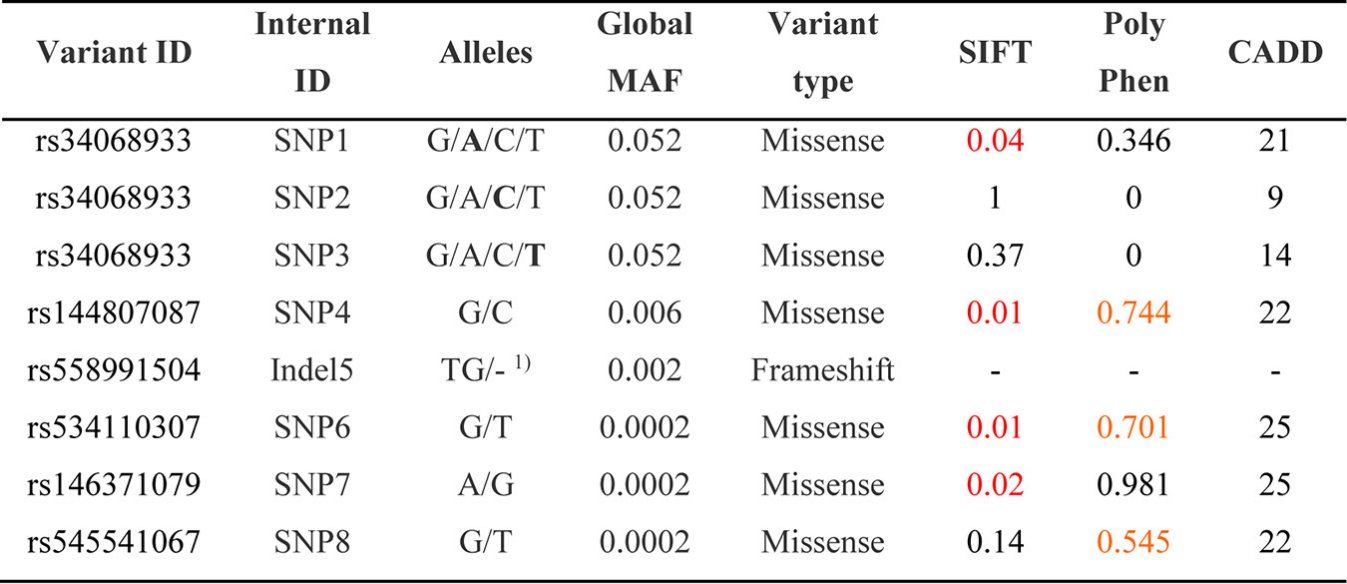
Overview of different analyzed mutants^*a*^

a SIFT, PolyPhen. and CADD bioinformatics scores predicting the deleteriousness of a given coding variant on protein function. Red, deleterious; orange, possibly damaging. Variants with CADD scores greater than 20 are within the top 1% of reference genome single nucleotide variants and thus have a high likelihood of changing protein function. Bold letters represent the variant allele of the given variant in case of the rs34068933 variants. —, scores are not available for indel variants. A third allele with a TG insertion was not included in this study.

### Human *CD302* transcript variants differ in their HCV restriction ability.

**FIG 5:**
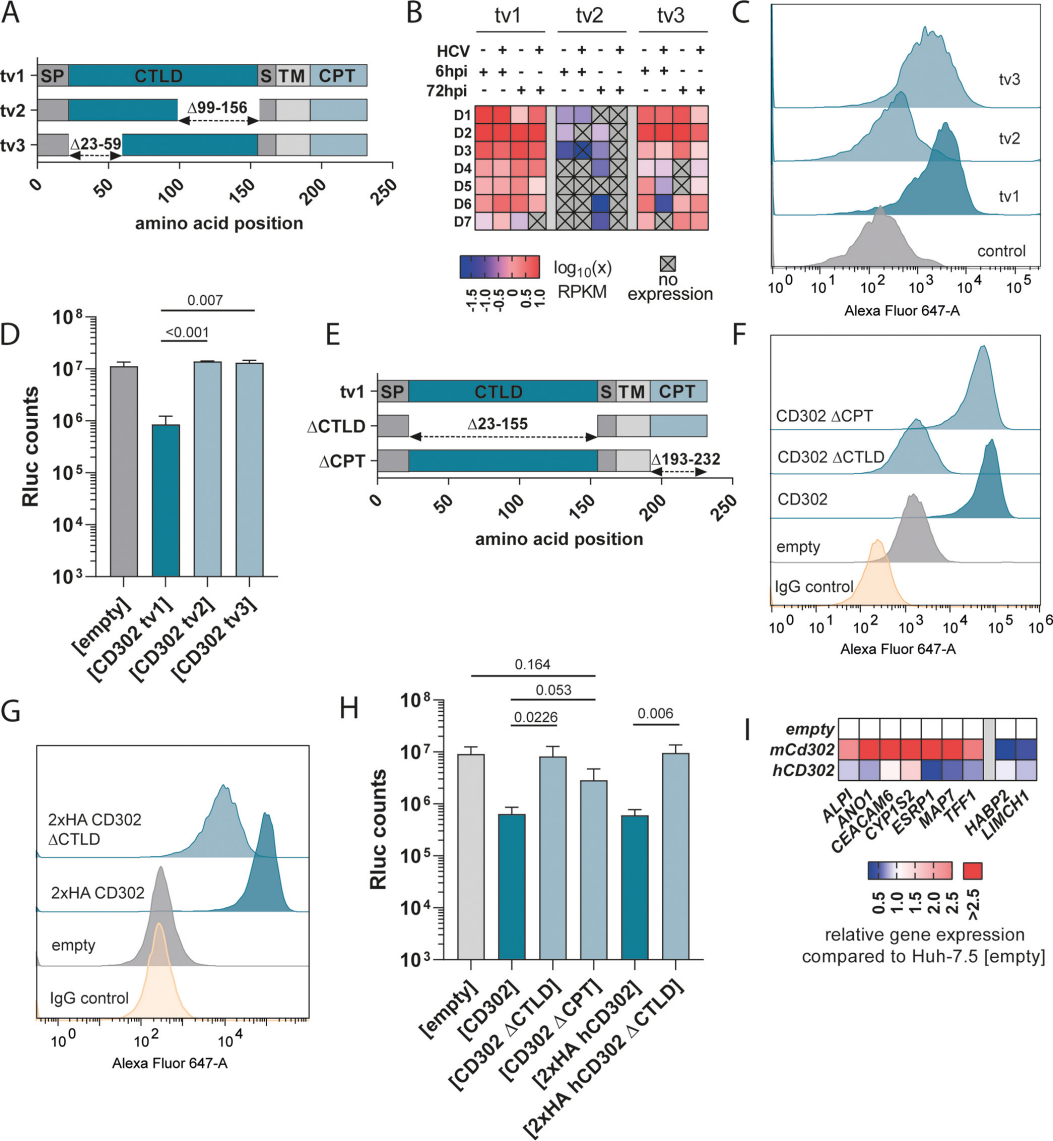
HCV restriction of *CD302* transcript variants and their expression in primary human hepatocytes. (A) Overview of encoded proteins from different *CD302* transcript variants (tv). Dashed arrows indicate missing amino acids as displayed above compared to the full-length protein (tv1). SP, signal peptide; CTLD, C-type lectin-like domain; S, spacer; TM, transmembrane domain; CPT, cytoplasmic tail. (B) Transcript expression levels in different PHH donors (D1 to D7) with or without HCV infection *ex vivo* (13). Plus signs above heatmap indicate whether a sample was infected with HCV and at which time point samples were taken for further analysis. (C and D) Transcript variants differently restrict HCV infection. Huh-7.5 cells overexpressing the different transcript variants were infected with GT2a J6 JcR2a for 72 h, and luciferase counts were measured. Overexpression of proteins in Huh-7.5 cells was measured by FACS. (E) Overview of protein composition of different deletion mutants. Dashed arrows indicate deleted amino acids as displayed above compared to the full-length protein (tv1). (F to H) Deletion of functional domains impact protein function. Either the CTLD or the CPT was deleted, and their restriction ability was compared to the WT protein upon ectopic expression in Huh-7.5 cells. HA-tagged variants were generated to confirm protein expression and functional relevance. Protein expression upon overexpression in Huh-7.5 cells was measured by FACS. Adjusted *P* values are indicated above bars and were determined by one-way analysis of variance (ANOVA) with Tukey test to correct for multiple comparisons. (I) Relative mRNA transcript dysregulation mediated by ectopic human *CD302* or murine *Cd302* expression. Cell lysates were collected from different Huh-7.5 cell passages ectopically expressing either an empty lentivirus, human *CD302*, or murine *Cd302*. Genes were selected based on their degree of dysregulation as previously reported (20). Changes in gene expression are relative to empty control cells and were determined via RT-qPCR and the 2^−ΔΔ^*^CT^* method.

In addition to genetic variation in the human population, the *CD302* gene also encodes four distinct transcript variants, three of which encode proteins (Fig. 5A). In our previous infection assays, we used transcript variant 1 (tv1), which encodes the full-length protein and consists of 232 amino acids. However, tv2 and tv3 contain in-frame deletions within the CTLD of the protein, leading to deletion of residues 99 to 156 for tv2 and 23 to 59 for tv3.

To examine functional properties of these variants and to judge their relevance *in vivo*, we first analyzed RNA- sequencing (RNA-seq) data of HCV-infected PHHs and found that all three transcript variants are expressed, albeit at different levels (Fig. 5B). Tv1 and tv3 were expressed in samples from all 7 donors, whereas tv2 was undetectable in at least one sample of each of the donors and generally exhibited very low mRNA expression (<0.5 reads per kilobase per million [RPKM]). As expected from our previous analysis, expression of neither variant was regulated upon HCV infection. When ectopically expressed in Huh-7.5 cells, only tv1 encoding the full-length CD302 protein restricted HCV infection (Fig. 5C and D), but not tv2 and tv3, suggesting an important functional role for the CTLD in HCV restriction.

To test functional relevance of the CTLD and the CPT, we created mutant constructs containing domain deletions and transduced them into Huh-7.5 cells (Fig. 5E). Deleting the CTLD completely abrogated HCV restriction, underpinning the importance of the CTLD for the function of CD302 as an HCV restriction factor. Of note, deletions of the CTLD prevented detection of the protein by our CD302-specific antibody (Fig. 5F). To confirm expression of CTLD-deleted constructs, we created a double hemagglutinin (HA)-tagged variant of CD302 and a CTLD-deleted CD302 variant. Both proteins were well detected at the cell surface using an HA tag-specific FACS assay (Fig. 5G). In accordance with the data from the untagged proteins, only the full-length protein restricted HCV (Fig. 5H). Ectopic expression of CD302 with deleted CPT also decreased HCV infection compared to the control vector-transduced cells. However, the extent of inhibition was less than when the full-length CD302 protein was expressed, suggesting that the CPT modulates the function of CD302 as restriction factor, but is not absolutely essential.

It was previously shown that coexpression of murine Cd302 (mCd302) and murine Cr1l enhanced HCV restriction (20) and induced specific transcriptional changes, which depended on the CPT of mCd302. To test if human CD302 modulates mRNA transcription in a similar manner to mCd302, we quantified the mRNA levels of several genes, which are up- or downregulated in Huh-7.5 cells ectopically expressing mCd302 (20). As expected, our reverse transcription-quantitative PCR (RT-qPCR)-based analysis confirmed the pronounced regulation of these genes by ectopic expression of mCd302, as, for instance, the mRNA level of *ESRP1* was increased more than 10-fold compared to the Huh-7.5 (empty) cells (Fig. 5I). In contrast, ectopic expression of CD302 had generally only modest effects on the mRNA levels of these genes (regulation less than 2-fold compared to Huh-7.5 (empty) cells. Moreover, the gene expression changes did not mirror the changes caused by ectopic expression of mCd302. For instance, the mRNA expression of *ESRP1* was upregulated by mCd302 but slightly downregulated by expression of CD302 (Fig. 5I). Taken together, these results show that CD302 and mCd302 transcriptional activity differs from each other both in extent and the spectrum of genes that are regulated.

### CD302 mediates HCV restriction at the cell surface.

**FIG 6:**
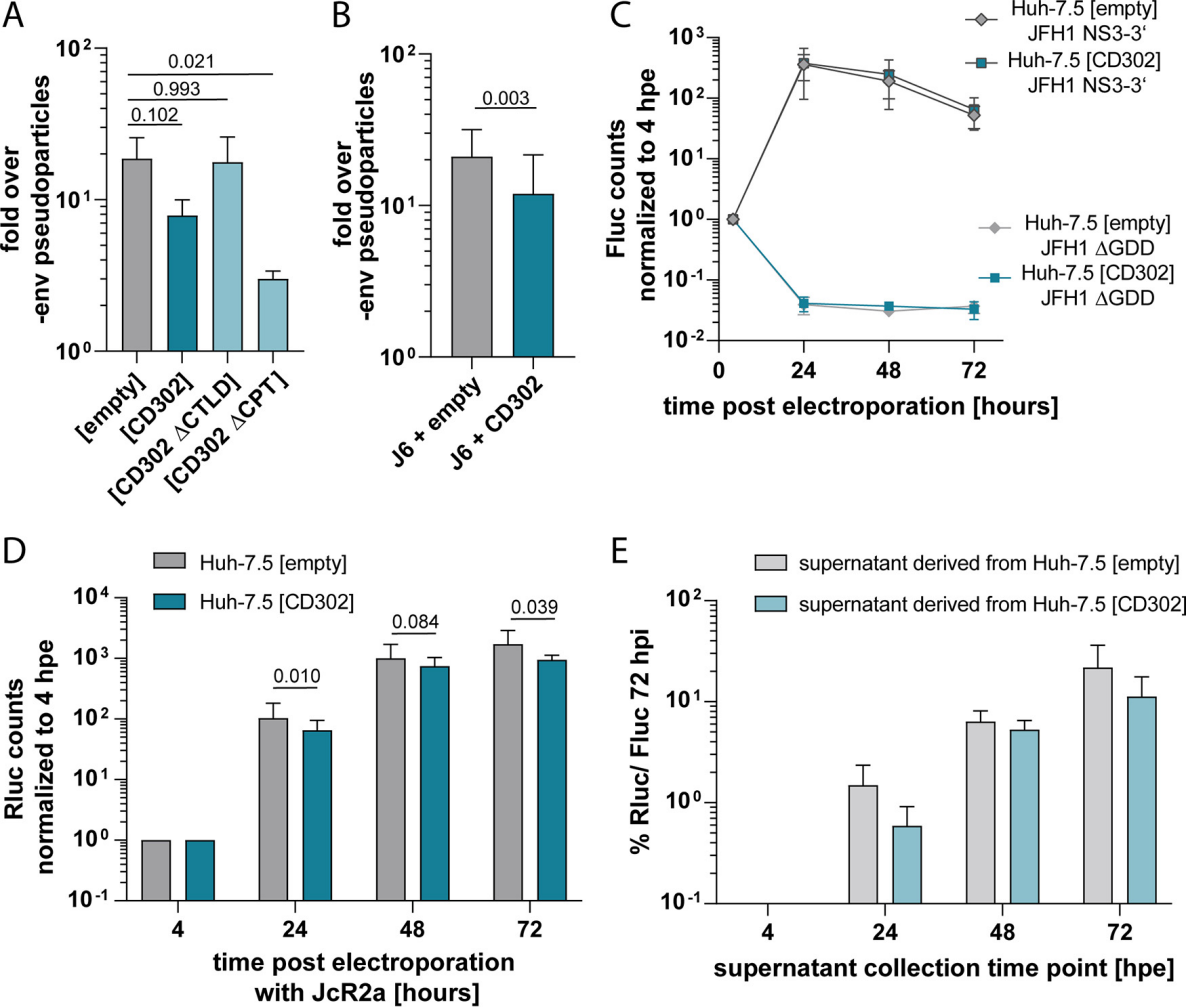
Effect of CD302 on different steps of the HCV life cycle. (A) Lentiviral pseudoparticles harboring J6 envelope proteins were produced and used to infect indicated overexpressing cell lines. At 72 hpi, firefly luciferase measurement was performed. Adjusted *P* values are indicated above the bars and calculated by one-way ANOVA with Dunnett’s test for multiple comparisons based on normalized values as presented in the graph. (B) For pseudoparticle production, HEK 293T cells were cotransfected with J6 E1E2 and CD302 or empty vector expression plasmids. Pseudoparticles were used to infect Huh-7.5 cells for 72 h. The *P* values are presented above bars and were determined by a one-tailed *t* test based on normalized values as presented in the graph. (A and B) Fluc counts were normalized to pseudoparticles produced with an empty vector (pcDNA/-env) instead of the J6 E1E2-encoding plasmid. (C) JFH1 subgenomic replicon or its replication-deficient ΔGDD mutant were electroporated into overexpressing Huh-7.5 cells and Fluc counts measured after 4, 24, 48, and 72 h postelectroporation (hpe). Fluc counts were normalized to 4-h time point. (D) Transcript RNA encoding full-length JcR2a was electroporated into Huh-7.5 (empty) and (CD302) cells, and half of the seeded cells were treated with 2′CMA inhibiting replication (data not shown). Cells were harvested at several time points post electroporation, and Rluc was measured. P-values are shown as determined by multiple t-test with correction for multiple comparisons (FDR (Q) < 0.05 only at 24 hpe). (E) Supernatants from electroporated cells were harvested at time points shown in panel D and used to infect Huh-7.5 (Fluc) cells for 72 h. (A to E) Results show 3 to 4 biological replicates.

To further elucidate the mechanism(s) by which CD302 restricts HCV, we examined its influence on HCV cell entry, translation, replication, and infectious virus production. We generated lentiviral pseudoparticles (HCVpps) harboring E1E2 of the J6 isolate (Gt2a) and infected Huh-7.5 vector control cells or Huh-7.5 cells with different CD302 variants (Fig. 6A). Ectopic expression of CD302 and CD302 ΔCPT consistently downregulated HCVpp infection. In contrast, HCVpps infected Huh-7.5 (CD302 ΔCTLD) as well as Huh-7.5 (empty) control cells with comparable efficiency. These results suggest that CD302 restricts HCV cell entry and that the CTLD is critical for this, whereas the CPT is dispensable.

Considering that CD302 inhibits HCVpp entry, possibly via direct interactions with HCV envelope proteins, we speculated that coexpression of CD302 during HCVpp production in transfected HEK 293T cells may limit production or infectivity of HCVpps. To test this, we cotransfected a CD302 expression plasmid or an empty control vector during production of HCVpps in HEK 293T cells and subsequently measured the infectivity of these particles in Huh-7.5 cells (Fig. 6B). Consistently between replicates, pseudoparticles produced together with the CD302 expression plasmid were less infectious than those produced when the empty control vector was cotransfected.

Next, we transfected subgenomic luciferase JFH1 replicon RNA into Huh-7.5 (CD302) or Huh-7.5 (empty) cells to quantify HCV RNA replication (Fig. 6C). To estimate a potential impact of CD302 expression on HCV RNA translation, we transfected a replication-defective replicon RNA with a deletion of the RNA polymerase GDD motif (ΔGDD) into the same set of cell lines. Notably, luciferase expression from both transfected replicons was essentially indistinguishable between these cell lines across the entire time course, suggesting that RNA translation and RNA replication of these replicon RNAs are not modulated by ectopic expression of CD302. In addition, we electroporated RNA of the full-length JcR2a reporter virus into the CD302-overexpressing cells and measured accumulation of luciferase activity in the transfected cells (Fig. 6D). We also collected culture fluids of these transfected cells and used them to inoculate naive Huh-7.5 (Fluc) cells (Fig. 6E). Accumulation of luciferase activity was slightly lower over time in transfected Huh-7.5 (CD302) cells compared to control cells. In parallel, we observed slightly less infectious virus production from these cells as is evidenced by somewhat reduced transduction of luciferase activity upon inoculation of Huh-7.5 (Fluc) cells.

### CD302 inhibits another liver tropic virus.

**FIG 7:**
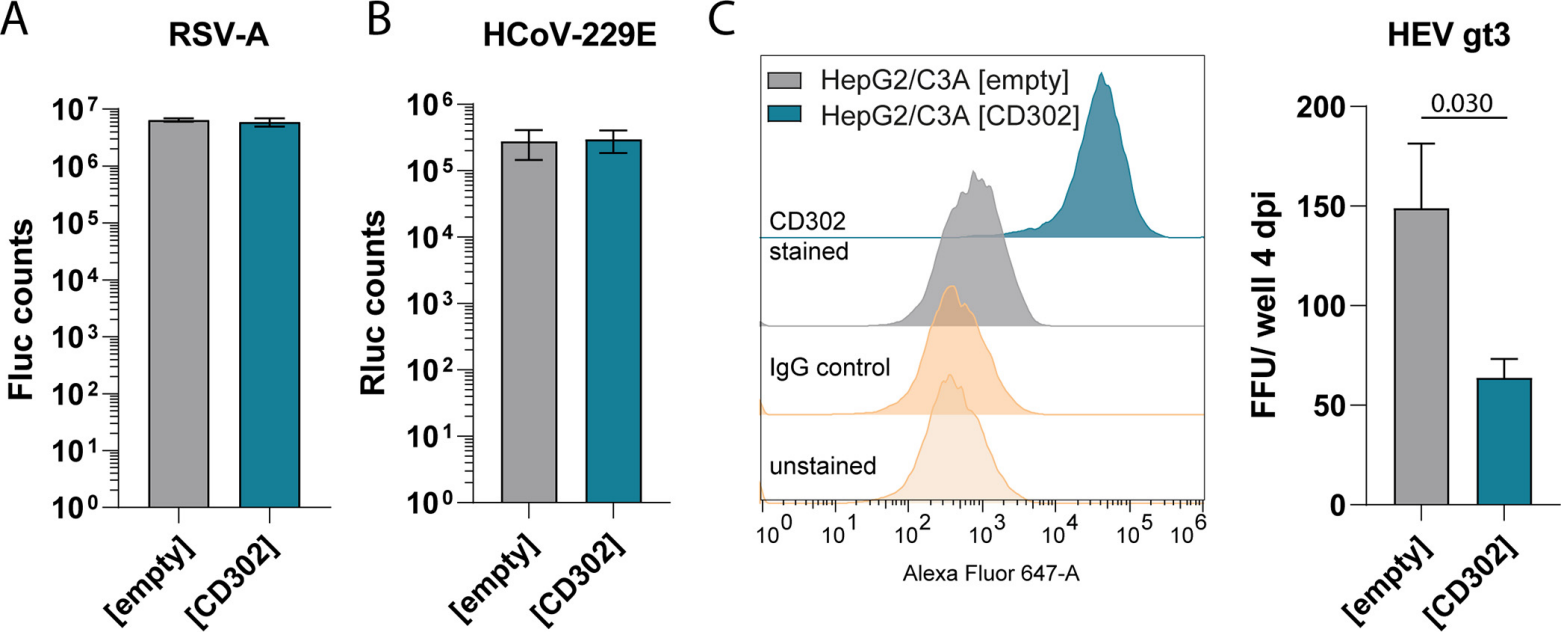
Influence of ectopic CD302 expression on other viruses. (A) Huh-7.5 overexpression cell lines were infected with a luciferase expressing RSV-A virus (51) at an MOI of 0.1 for 4 h. Forty-eight hours postinfection, cells were lysed, and Fluc counts were measured. Results from three independent experiments are given. (B) Huh-7.5 (empty and CD302) cells were infected with human coronavirus 229E (HCoV-229E) for 48 h until luciferase measurement. The mean and SD of three biological replicates are shown. (C, Left) Protein expression in HepG2/3CA cells either expressing *CD302* or an empty vector control. (C, Right) Corresponding HEV genotype (gt) 3 infection measured as focus-forming units (FFU) per well. The graph shows three biological replicates with mean and SD. The *P*-value is indicated above the bars and was determined by a two-tailed *t* test. dpi, days post infection.

To evaluate whether *CD302* specifically restricts HCV infection or has a broader antiviral effect also targeting other viruses, we infected Huh-7.5 (CD302) cells with human respiratory syncytial virus (RSV-A) (Fig. 7A) and human coronavirus 229E (HCoV-229E) (Fig. 7B). Both respiratory viruses infected Huh-7.5 cells at similar rates irrespective of *CD302* overexpression. Additionally, we generated HepG2/C3A cells ectopically expressing either CD302 or an empty control vector and confirmed CD302 protein expression via FACS staining (Fig. 7C, left). Both stable cell lines were infected with a hepatitis E virus (HEV) genotype 3, a liver tropic virus. Four days after infection, fewer focus-forming units were observed in HepG2/C3A (CD302) cells than HepG2/C3A (empty) cells (Fig. 7C, right), suggesting that CD302 reduced HEV infection in this cellular model.

## DISCUSSION

In this study, we show that endogenously and ectopically expressed human *CD302* restricts HCV infection. Among the transcript variants expressed in primary human hepatocytes, only the most abundant variant (tv1), encoding the full-length CD302 protein, restricted HCV in our experimental setup. Transcript variants 2 and 3, both of which were carrying in-frame deletions of parts of the CTLD, were nonfunctional in the HCV infection assay. Congruently, deletion of the entire CTLD of *CD302* ablated HCV restriction, underpinning that the CTLD is essential for the functioning as HCV restriction factor. Additionally, we found that naturally occurring coding polymorphisms within *CD302* are relatively rare, with minor allele frequencies of 0.052 and lower. All CD302 variants with protein coding changes, including those with SIFT and PolyPhen scores predictive of high deleteriousness, maintained their capacity to restrict HCV, even the ones located in the CTLD. Thus, the anti-HCV function of *CD302* in human liver cells is likely conserved across diverse human populations. Notably, indel 5 (rs558991504) results in the production of a truncated CD302 protein lacking 20 residues of the CPT, which, however, did not ablate HCV restriction. In line with this, complete deletion of the CPT reduced the capacity of the protein to inhibit HCV infection but did not fully ablate it. Moreover, the CD302 ΔCPT construct retained an antiviral activity also in the HCVpp cell entry assay. Taken together, these results indicate that the CTLD of *CD302* is essential for HCV restriction, while the CPT is dispensable but modulates the extent of HCV restriction.

In general, these findings are in agreement with our previous report describing mouse *Cd302* as an HCV restriction factor which limits infection of mouse hepatocytes (20). However, in the case of m*Cd302*, we observed that its ectopic expression in human hepatoma cells induced more than 100 genes, including 88 IFN-regulated genes and metabolic genes downstream of nuclear signaling. Regulation of these genes depended on presence of the cytoplasmic tail of the protein and correlated with its antiviral function. We tested a representative subset of 9 genes representing the transcriptional program occurring in Huh-7.5 (mCd302) cells, but their expression changes in Huh-7.5 (CD302) cells were minimal or even in the opposite direction.

In the case of *mCD302*, gene knockout in mouse hepatocytes mirrored the transcriptional changes of overexpressed *mCd302*, and pharmacological inhibition of JAK/STAT signaling did not ablate the antiviral activity of mouse *Cd302*. Similar to the latter observation, we detected enhanced HCV infection in PHHs upon *CD302* silencing in the presence of ruxolitinib, a JAK/STAT inhibitor. This suggests that both the mouse and human proteins modulate HCV infection independent of IFN signaling. The high level of conservation at the genetic level and very prominently across the CTLD structure suggests that these proteins may inhibit HCV infection by common mechanisms (20). However, there are also differences: murine Cd302-mediated HCV restriction and transcriptional changes were enhanced by coexpression of mouse *Cr1l*, a murine complement component (3b/4b) receptor, which is also expressed in mouse liver cells (20). In contrast, humans encode a distinctly longer CR1l protein, which, however, does not restrict HCV and is not expressed in human hepatocytes (20). Thus, there may be different cofactor requirements between these species, and overall, the *CD302*-dependent restriction may be less pronounced in human hepatocytes, as *Cr1l* is not coexpressed.

The CD302 protein was primarily detectable at the cell surface. Additionally, mechanistic analysis showed no effect on RNA translation and RNA replication of HCV replicons. Contrastingly, we noted a slight effect on replication and infectious virus production of a luciferase reporter virus genome. This suggests either a modest impact on replication of HCV full-length RNAs and/or a minor effect on cell-to-cell spread and/or virus release. Our experiments cannot distinguish between these potential effects. However, given the modest effect size, we believe that these mechanisms are not primarily responsible for the restriction of HCV by CD302. Susceptibility of Huh-7.5 cells ectopically expressing CD302 to HCV from diverse genotypes was reduced up to 18-fold. Combined with the finding that CD302 is expressed at the cell surface and that it reduced cell entry of HCVpps suggests that it primarily inhibits HCV at the cell entry stage. In this context, it is notable that production of HCVpps in the presence of CD302 slightly reduced their infectivity. While we did not explore the mechanisms of inhibited HCVpp infectivity in the latter setting, it is possible that a direct binding between HCV envelope proteins E1 and E2 and CD302 may reduce particle production or their infectivity. In the case of overexpressed *mCd302*, we previously noted reduced trafficking of fluorescently labeled HCVcc particles to tight junctions of Huh-7.5 cells (20). Such an aberrant trafficking of HCV particles may be caused by a direct interaction between HCV envelope proteins and *mCd302.* Such interactions may competitively inhibit interactions with HCV cell entry factors such as SR-B1 and CD81 and prevent particle trafficking and, in turn, cell entry. In the case of mCd302 overexpressed in CHO-745 cells, we observed a slightly enhanced HCVcc binding compared with parental CHO-745 cells, supporting the notion that components of HCV particles may bind to mCd302. Considering these observations made with the murine orthologue of CD302, the cell entry defect upon expression of CD302 may also be caused by interactions between HCVcc particle components and CD302. Clearly, more work is needed to pinpoint if such interactions occur also in the case of CD302, which particle components may be important for such interactions, and if these are critical for the restriction of HCV infection.

Interestingly, we found that ectopically expressed CD302 also inhibits HEV infection but that it does not modulate RSV or HCoV-229E infection. These results suggest that CD302 may modulate cellular susceptibility to a selected spectrum of viruses. While we have not explored the antiviral mechanism against HEV, these data encourage future research into which other hepatotropic viruses may be affected by CD302 expression and via which mechanism(s) this may occur.

*CD302* is primarily expressed in the liver, followed by lung tissue and peripheral blood leucocytes (21). It belongs to the group of CTLDcps, which is made up of a large superfamily of metazoan proteins with diverse functions (35). Expression of CTLDcps at the surface of myeloid cells is essential for innate and adaptive immune responses. They recognize pathogen-associated glycolipids or glycoproteins via their CTLD and trigger responses such as endocytosis, phagocytosis, and pro- or anti-inflammatory reactions (36, 37). In dendritic cells (DCs) and macrophages, they coordinate signaling cascades regulating inflammatory cytokines, including interleukin-6 (IL-6), tumor necrosis factor alpha (TNF-α), and others, thereby mediating innate and adaptive immune responses (38). Viruses, including HIV-1, HCV, and dengue virus (DENV), subvert CTLDcp functions, with implications for virus transmission and infection outcome (38). For instance, dendritic cell-specific ICAM-grabbing nonintegrin (DC-SIGN) on DCs captures HIV-1 particles facilitating transmission and dissemination by *trans*-infection of CD4-positive (CD4^+^) T cells (38–40). DC-SIGN also interacts with other viruses, including HCV. HCV bound to DC-SIGN traffics to nonlysosomal compartments of dendritic cells, which protects it from degradation, possibly facilitating transmission to hepatocytes (41). Little is known about how CTLDcps contribute to liver-intrinsic immunity in humans. The immunoprivileged environment of the liver may require dedicated CTLDcps to mount an extra layer of intrinsic immunity for pathogen defense. Although *CD302* alone is certainly not sufficient to prevent HCV or HEV infection, it is a novel CTLDcp which likely contributes to human liver-intrinsic antiviral defenses. It will be interesting to explore the effect of *CD302* on other hepatotropic viruses as well and investigate the mode of action in more detail.

## MATERIALS AND METHODS

### Culturing of cell lines.

Huh-7.5 and HEK 293T cells were cultured in Dulbecco’s modified Eagle’s medium (DMEM) supplemented with 1% nonessential amino acids (NEAA), 2 mM l-glutamine (all Gibco, Thermo Fisher Scientific, Waltham, MA, USA), and 10% fetal calf serum (FCS) (Capricorn Scientific GmbH, Ebersdorfergrund, Germany). HepG2/C3A cells were cultured in minimum essential medium (MEM) supplemented with 8.8% ultralow IgG FCS, 2 mM l-glutamine, 1% NEAA, 1 mM sodium pyruvate, and 100 μg/mL gentamicin sulfate (all Gibco, Thermo Fisher Scientific). The plates were coded with phosphate-buffered saline (PBS) containing 0.1% acetic acid and 0.01% collagen (Serva, Heidelberg, Germany).

Plated PHHs were provided by the Department of General, Visceral, and Transplant Surgery at Hannover Medical School. Hepatocytes were isolated from liver tissue from donors undergoing partial hepatectomy with written informed consent approved by the Ethics Commission of Hannover Medical School (Ethik-Kommission der MHH; no. 252-2008). PHHs were cultured as previously reported (42). Additionally, PHHs were commercially obtained (Lonza, Basel, Switzerland). Cryopreserved PHHs were thawed as recommended by the manufacturer. Briefly, hepatocytes were placed in a water bath at 37°C for 90 to 120 s and transferred into 50 mL of warmed thawing medium (catalog no. MCHT50; Lonza). After centrifugation, supernatant was aspirated off without detaching the cells, resuspended in plating medium (catalog no. Lonza), and seeded into collagen-coated 6-well dishes. All PHHs were cultured in supplemented HCM medium (catalog no. CC-3198; Lonza).

### Generation of stably overexpressing cell lines.

Previously cloned plasmids containing the human *CD302* sequence and encoding a blasticidin or puromycin resistance gene (20) were used as the template in a PCR-based cloning approach to generate sequences containing deletions or point mutations that were described in this study. PCRs were performed using Q5 high-fidelity DNA polymerase (NEB, Ipswich, MA, USA) according to the manufacturer’s instructions with specifically designed primers. After verifying their length by agarose gel electrophoresis, PCR products were purified with NucleoSpin gel and PCR clean-up (Macherey-Nagel, Düren, Germany) and inserted into the lentiviral vector pWPI (Addgene, Watertown, MA, USA) via restriction cloning. Correct nucleotide sequences of inserts were confirmed by Sanger sequencing (GATC, Konstanz, Germany). Equimolar amounts of vesicular stomatitis virus envelope (VSV-G), HIV-1gag/pol, and the respective pWPI plasmid were transfected into HEK 293T cells using Lipofectamine 2000 (Thermo Fisher Scientific) and supernatants harvested at 24 and 48 h post-sodium butyrate induction. Supernatants were filtered using 0.45-μm cellulose acetate membrane filters (VWR, Radnor, PA, USA) and pooled before transduction of 2 × 10^5^ Huh-7.5 cells, Huh-7.5 (Fluc) cells, or HepG2/C3A cells. After 72 h, cells were selected with 5 μg/mL blasticidin (Invivogen, San Diego, CA, USA) or 2.5 μg/mL puromycin and expanded.

### HCV production and detection.

*In vitro* transcription of HCV isolates was generated as previously described (43). RNA integrity and concentration were checked by spectrophotometry and agarose gel electrophoresis. Huh-7.5.1 cells were electroporated with HCV genomic RNA transcripts (wild-type Jc1 chimera and Renilla luciferase reporter viruses [Con1/1b/R2a, Jc1R2a, S52/3a/R2a, ED43/4a/R2a, SA13/5a/R2a, HK6a/6a/R2a, and QC69/7a/R2a]) (Table 2) (28, 44, 45). Supernatants were harvested at 48, 72, and 96 h postelectroporation, filtered (0.45-μm cellulose acetate membrane filters [VWR]), pooled, aliquoted, and frozen at −80°C. Virus infectivity was tested with limiting dilution assays to determine 50% tissue culture infective dose (TCID_50_)/mL (46, 47) or by measuring luciferase activity after 72 h on Huh-7.5 cells.

**TABLE 2:** Luciferase-expressing HCV chimeras used in this study

Short name	Long name	GenBank accession no.
GT1b (Con1)	Con1/1b/R2a	MT955903
GT2a (J6)	JcR2a	MT955904
GT3a (S52)	S52/3a/R2a	MT955911
GT4a (ED43)	ED43/4a/R2a	MT955912
GT5a (SA13)	SA13/5a/R2a	MT955913
GT6a (HK6a)	HK6a/6a/R2a	
GT7a (QC69)	QC69/7a/R2a	

### Flow cytometry.

Cells were trypsinized, pelleted, and resuspended in FACS buffer (1% FCS in PBS). Cells were treated with PBS containing 1% FCS and 0.5% saponin for 20 min on ice for permeabilization. Unconjugated monoclonal antibody specific for CD302 (human CD302/CLEC13A; R&D Systems; monoclonal mouse immunoglobulin G1 [IgG1]) or a mouse IgG1 isotype control (R&D Systems, Minneapolis, MN, USA) were diluted in FACS buffer or permeabilization buffer to 5 μg/mL and added to cells for 30 min at 4°C. Following two washes with FACS buffer, a specific anti-mouse secondary antibody conjugated to Alexa Fluor 488 or Alexa Fluor 647 (Thermo Fisher Scientific) was diluted 1:1,000 in FACS buffer or permeabilization buffer and added to cells. After three washes with FACS buffer, stained cells were resuspended in FACS buffer or fixation buffer (1% FCS and 0.5% paraformaldehyde [PFA] in PBS) and analyzed by BD FACSCalibur (BD Biosciences, Franklin Lakes, NJ, USA) or SONY SA3800 spectral analyzer. Data were analyzed using FlowJo software (BD Biosciences).

### Immunofluorescence.

Huh-7.5-overexpressing cells were seeded on glass coverslips, fixed with 3% PFA at room temperature for 10 min on the following day, and washed twice with PBS. Subsequently, cells were treated with PBS containing 0.1% Triton X-100 for 10 min at room temperature to permeabilize. After washing twice with PBS, coverslips were incubated in a 5% solution of goat serum in PBS for 1 h at room temperature to block potential nonspecific antibody binding. For CD302 detection, human CD302/CLEC13A mouse monoclonal antibody (R&D Systems) was diluted to 5 μg/mL and incubated at room temperature for 1 h or overnight at 4°C. Afterward, coverslips were washed three times with PBS, followed by an incubation with anti-mouse secondary antibody conjugated to Alexa Fluor 488 (Thermo Fisher Scientific) in a 1:1,000 dilution at room temperature in the dark for 1 h. After washing again twice with PBS, the nuclei were stained using a 0.5 μg/mL 4′,6-diamidino-2-phenylindole (DAPI) solution in water. Finally, after one more washing step with water, mounting of the coverslips in ProLong Gold antifade mountant (Thermo Fisher Scientific) was performed, and they were left to dry overnight at room temperature in the dark. Images were taken with an inverted confocal laser-scanning microscope (Olympus FluoView 1000) with FluoView 1000 imaging software (Olympus, Tokyo, Japan).

### RNA-seq analysis and data mining.

Single-cell data were analyzed by Aizarani et al. and gene expression visualized using their website, http://human-liver-cell-atlas.ie-freiburg.mpg.de/ (31). Gene expression data of liver biopsy specimens were downloaded from GEO under accession number GSE84346 submitted by Boldanova et al. (32) and plotted with GraphPad Prism 9. Analyses of initial transcriptional response to HCV in PHHs and Huh-7.5 were conducted in a separate study, and data were submitted to GEO (accession number GSE166428) (13). In summary, cells were infected with Jc1 chimera and cellular RNA isolated after 6 and 72 h. Samples were run on the Illumina HiSeq 2500 platform. Mapping, gene, and transcript variant expression analyses were conducted with CLC Workbench (Qiagen, Aarhus, Denmark).

The protein structure of CD302 was predicted using the corresponding webpage (https://alphafold.ebi.ac.uk/) with the AlphaFold model (48).

### HCV infection assays.

We seeded 1 × 10^4^ Huh-7.5 cells overexpressing *CD302*, its mutants, or an empty vector control per well in 96-well plates. On the following day, cells were infected, and medium was changed 4 h later and incubated for 72 h. To analyze luciferase activity, cells were washed once with PBS and lysed with 35 μL water, and plates were frozen down at −80°C until they were measured with a plate luminometer (Berthold, Bad Wildbad, Germany) and coelenterazine (Carl Roth GmbH & Co. KG; in methanol) diluted 1:1,000 in PBS.

### Replication assays and virus particle release.

Subgenomic replicon (SGR) constructs (based on pFK_i389Luc_NS3-3′_JFH_dg-delta GDD and pFKi389_F-Luc_EI-NS3-3′/JFH1 plasmids) and *Renilla* luciferase reporter virus JcR2a were *in vitro* transcribed (43). We electroporated 6 × 10^6^ Huh-7.5 cells either overexpressing CD302 or the empty vector control with 5 μg of RNA transcripts as mentioned above. Subsequently, cells were seeded in triplicates in a 96-well format for the SGR assay. Replication of the SGR was investigated at 4, 24, 48 and 72 h postelectroporation. For that cells were lysed in 35 μL of a lysis buffer containing 1% Triton X-100, 25 mM Gly-Gly, 15 mM MgSO_4_, and 4 mM EGTA in water before luciferase activity measurement was performed. Full-length virus-electroporated cells were seeded in 6-well format in a density of 6 × 10^5^ cells per well, and 25 μM replicase inhibitor 2′ C-methyl adenosine (2′CMA), kindly provided by Timothy Tellinghuisen, was added to half of the seeded wells for both cell lines as a control. A medium change, including an additional application of 2′CMA, was performed at 4 h postelectroporation. To analyze virus particle release, supernatants were harvested at 4, 24, 48, and 72 h postelectroporation, followed by a second round of infection with collected supernatants of naive Huh-7.5 cells expressing the firefly luciferase in a 96-well format for 72 h. Replication of full-length virus in the electroporated cells at 4, 24, 48, and 72 h postelectroporation, and infection rate of the second round of infection was analyzed by luciferase activity measurement. For that, cells were washed once with PBS before cell lysis was performed by addition of 350 μL and 50 μL water, respectively, and a freezing step at −80°C until measurement. Measurement of the luciferase activity was performed by the utilization of a plate luminometer (Berthold, Bad Wildbad, Germany).

### HCV pseudoparticle assay.

Lentiviral pseudoparticles were generated with HCV E1E2 surface glycoproteins (isolate J6, genotype 2a), an empty vector as negative control (pcDNA empty), VSV-G as positive control, or J6 E1E2 together with pWPI empty or pWPI CD302 plasmids. Packaging construct pCMV-dR8.74 encoding gag/pol was cotransfected with the lentiviral vector (pWPI F-Luc) and the individual envelope expression constructs using polyethylenimine (PEI) (Polyplus, Illkirch, France). After 24 h, sodium butyrate (10 mM) was added to transfected cells and incubated for 6 h. Twenty-four hours after sodium butyrate induction, supernatant was collected, filtered through a 0.45-μm-pore-size filter (VWR International, USA), and used to inoculate target cells in 12-well plates for 72 h. Cells were washed with PBS and lysed in 250 μL lysis buffer (1% Triton X-100, 25 mM Gly-Gly, 15 mM MgSO_4_, 4 mM EGTA, and 1 mM dithiothreitol [DTT]) per well and transiently stored at −20°C. Firefly luciferase expression of 20 μL thawed lysates was measured using a plate luminometer (Microplate Reader Centro XS3 LB 960; Berthold) and d-luciferin (PJK Biotech, Kleinblittersdorf, Germany).

### RSV infection assay.

Huh-7.5 cells stably overexpressing CD302 or an empty vector control were seeded at a density of 2 × 10^4^ in a 96-well format the day before inoculation with an RSV-A luciferase reporter virus at a multiplicity of infection (MOI) of 0.1. Four hours after inoculation, virus inoculum was replaced by fresh medium, and cells were incubated for 48 h at 37°C and 5% CO_2_. Subsequently, cells were washed twice with PBS and lysed in 50 μL lysis buffer (1% Triton X-100, 25 mM Gly-Gly, 15 mM MgSO_4_, 4 mM EGTA, and 1 mM DTT) per well and transiently stored at −20°C. Luciferase expression of thawed lysates was measured using a plate luminometer (Microplate Reader Centro XS3 LB960; Berthold) and d-luciferin (PJK Biotech). Cell lysates were prediluted 1:20 in lysis buffer before measurement.

### HCoV-229E infection assay.

Huh-7.5 cells stably overexpressing CD302 or an empty vector control were seeded at a density of 2 × 10^4^ per well in a 96-well format and incubated overnight at 37°C with 5% CO_2_. Twenty-four hours postseeding, the cells were infected with 1,364 TCID_50_/well HCoV-229E *Renilla* luciferase reporter virus and incubated at 33°C and 5% CO_2_ for 48 h. Subsequently, cells were washed with PBS, treated with 50 μL lysis buffer (0.5% Triton X-100 in PBS) per well, and stored at −80°C. Renilla luciferase signal of thawed samples was measured using a plate luminometer (Microplate Reader Centro XS3 LB960) and coelenterazine (Carl Roth GmbH & Co. KG; in methanol) diluted 1:1,000 in PBS.

### HEV infection assay.

For infectious viral particle production, 9 × 10^6^ HepG2/C3A cells were transfected with 10 μg RNA of the hepatitis E virus genotype 3 Kernow C1 p6 viral strain, harboring the G1634R mutation in its RNA-dependent RNA polymerase, as previously described (49). Intracellular viral particles were harvested after 4 days in PBS; cells were lysed by 3 freeze-thaw cycles. For infection, 2 × 10^4^ HepG2/C3A cells overexpressing CD302 or empty vector control were seeded per well in a 96-well plate format. Cells were infected the following day with 30 focus-forming units (FFU)/well, and the infection was stopped after 4 days of incubation. After three washes with 100 μL PBS/well, cells were fixed using 4% paraformaldehyde solution for 10 min at room temperature, washed again thrice, and permeabilized for 5 min using PBS supplemented with 0.2% Triton X-100. Following blocking with 5% horse serum in PBS for 1 h at room temperature, a rabbit polyclonal serum immunized with HEV’s capsid protein, open reading frame 2 (ORF-2), diluted 1:5,000, was incubated on the cells overnight at room temperature. Cells were again washed and incubated with goat anti-rabbit Alexa Fluor 488 antibody (Thermo Fisher Scientific) diluted 1:1,000 for 2 h at room temperature. Focus forming units were counted manually using the EVOS M500 microscope.

### Silencing of *CD302*.

Cells were transfected with Lipofectamine RNAiMAX transfection reagent (Thermo Fisher Scientific) according to the manufacturer’s instructions in Opti-MEM (Gibco, Thermo Fisher Scientific). Endoribonuclease-prepared small interfering RNA (esiRNA) targeting EGFP as control and *CD302* (MISSION esiRNA; Sigma-Aldrich) were utilized for gene silencing in Huh-7.5 cells and PHHs. Experiments with Huh-7.5 cells, 1 × 10^4^ cells seeded per well, were conducted at a final concentration of 1 pmol per siRNA in 96-well plates, whereas experiments in PHHs were done in 6-well dishes at a final concentration of 25 pmol and medium containing 10 μM ruxolitinib (AdipoGen Life Sciences, San Diego, CA, USA). Silencing was performed 48 h before infection with HCV for 48 (Huh-7.5 cells) or 72 h (PHHs). Medium was changed 4 or 6 h postinfection with HCV. Supernatants of 6-cm dishes were collected, and PHHs were lysed with 350 μL of RA1 buffer per well supplemented with 1% β-mercaptoethanol (Macherey-Nagel). Samples were frozen at −80°C until further analyses. To measure luciferase counts, 96-well plates were handled in the same way as described above for other infection assays.

### RT-qPCR.

Total RNA from cell lines or PHHs were isolated using a NucleoSpin RNA kit (Macherey-Nagel) according to the manufacturer’s recommendations, and liver tissue was homogenized and total RNA extracted using TRIzol reagent (Invitrogen). Concentrations were measured by NanoDrop spectrophotometer (VWR). One hundred to 1,000 ng total RNA was reverse transcribed into cDNA using a PrimeScript first-strand cDNA synthesis kit (TaKaRa, Shiga, Japan). Subsequently, qPCR of cDNAs was performed using SYBR master mix (TaKaRa) and run on the LightCycler 480 (Roche, Basel, Switzerland). For normalization, glyceraldehyde-3-phosphate dehydrogenase (*GAPDH*) was used as the reference gene. To determine relative gene expression, the threshold cycle (2^−ΔΔ^*^CT^*) method (50) was applied. Tenfold serial dilutions of plasmids containing the *CD302* ORF were coamplified in the RT-qPCR cycler to generate a standard curve for absolute quantification (10^8^ to 10^1^ copies). Validated primer-pairs for RT-qPCR were taken from PrimerBank (https://pga.mgh.harvard.edu/primerbank/).
